# Culture of attached and suspended *Rhodopseudomonas faecalis* in the presence of decomposing fish feed

**DOI:** 10.1002/mbo3.924

**Published:** 2019-09-04

**Authors:** Xiaodong Wang, Xingguo Liu, Shimin Lu, Chong Liu, Zhaojun Gu, Xianlei Zeng, Qi Ni

**Affiliations:** ^1^ Fishery Machinery and Instrument Research Institute Chinese Academy of Fisheries Sciences Shanghai China

**Keywords:** attached bacteria, biofilms, fish feed, photosynthetic bacteria, *Rhodopseudomonas*

## Abstract

An approach to culturing attached and suspended forms of *Rhodopseudomonas faecalis* by using compound fish feed with tap water in transparent containers is reported in this study. The ratio of fish feed to tap water was 14.3–50.8 g/L, and no other inoculum or substances were added during the culture process. When the ratio of fish feed to tap water was 14.3 g/L, the highest total nitrogen, total phosphorus, and total dissolved carbon content recorded in the water in the containers were approximately 730 mg/L, 356 mg/L, and 1,620 mg/L, respectively, during the process of feed decay. *Comamonas, Rhodopseudomonas,* and *Clostridium* successively dominated during the culture process. *Rhodopseudomonas* was the most common dominant genus in both the attached and suspended forms when the water was dark red, and the relative operational taxonomic unit abundance reached 80‒89% and 24.8%, respectively. The dominant species was *R. faecalis*. The maximum thickness of attached bacteria and the biomass of attached *Rhodopseudomonas* reached up to 0.56 mm and 7.5 mg/cm^2^, respectively. This study provides a method for the mass culture of *Rhodopseudomonas* by using the fermentation of aquatic compound fish feed.

## Graphical abstract

Culture of attached and suspended forms of *Rhodopseudomonas faecalis* by using compound fish feed with tap water in transparent containers was achieved in a greenhouse. When the ratio of fish feed to tap water was 14.3 g/L during the culture, the maximum thickness of attached bacteria and the increased biomass of attached *Rhodopseudomonas* produced reached up to 0.56 mm and 7.5 mg/cm^2^, respectively.

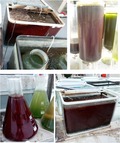

## 1 INTRODUCTION

Photosynthetic bacteria (PSB) are strains of bacteria with light‐absorbing pigments, which convert light energy into chemical energy and play an important role in the circulation of material, energy conversion, and water purification in aquatic ecosystems (Madigan & Jung, 2009). *Rhodopseudomonas*, a member of the purple, nonsulfur, photosynthetic bacteria (PNSB), is facultatively anaerobic, Gram‐negative and rod‐shaped. It is capable of nitrogen and carbon dioxide fixation as well as photosynthesis. The presence of carotenoids makes *Rhodopseudomonas,* a well‐known genus of purple phototrophic bacteria (PPB, largely consisting of PNSB), which range from orange to purple in color.

Many PPB species have been studied, and their applications have been extensively investigated (Loo, Vikineswary, & Chong, 2013; Saejung & Thammaratana, 2016), including those of *Rhodopseudomonas* species, which are cultured for aquaculture feed (Kim & Lee, 2000) and have been suggested to be a high carotenoid producer (Saejung & Apaiwong, 2015) and a hydrogen producer (Liu, Xie, Guo, Ding, & Ren, 2011; Ren et al., 2012; Xie et al., 2012). PNSB play primary roles in wastewater treatment processes (Okubo, Futamata, & Hiraishi, 2005), whereas PPB have been used for the treatment of organic wastewaters from the sugar, food processing, chicken abattoir, dairy, and fermented starch industries.

Some reports have indicated the dominance of PNSB in both the suspended and attached forms. *Rhodopseudomonas* species are found in various environments including limnetic zones, marine habitats, and sewage sludge, which are particularly abundant in eutrophic environments (Wei, Okunishi, Yoshikawa, Kamei, & Maeda, 2016; Zhang, Yang, Huang, Zhang, & Liu, 2002). Mixed communities of anoxygenic PPB and Chlorella‐type green algae have been reported to dominate leachate sewage ponds, which are typical hypereutrophic red water ponds (Chandaravithoon, Nakphet, & Ritchie, 2018). Some pink microbial mats have been found to naturally occur in a swine wastewater ditch, in which the microbial mats were dominated by the genera *Rhodobacter* and *Rhodopseudomonas,* and the *Rhodopseudomonas* species was more dominant than the *Rhodobacter* species (Okubo, Futamata, & Hiraishi, 2006). A continuous photo‐anaerobic membrane reactor started with raw domestic wastewater has been found to change to purple with PPB dominance during culture, and *Rhodocyclus* or *Rhodopseuodomonas* and *Rhodobacter* or *Rhodobacter* dominance have been found to occur during different culture stages (Hülsen et al., 2016).

In photofermentation H_2_ production research, bacterial immobilization technology resulting in biofilm formation with PNSB dominance has been used to enhance photo‐H_2_ production (Levin, Pitt, & Love, 2004; Mohan et al., 2007; Robledo‐Ortíz, Ramírez‐Arreola, Gomez, González‐Reynoso, & González‐Núñez, 2010; Tian et al., 2010) with a synthetic culture medium. Chemical synthetic media are usually used for the culture of *Rhodopseudomonas* (Weaver, Wall, & Gest, 1975). Models of PSB biofilm formation have been developed, considering bacterial inactivation, light intensity, pH values and initial inoculation, in the construction of biofilm photobioreactors (Chen, Pu, Liao, Zhu, & Wang, 2013). However, no reports have described the culture of attached PPB such as *Rhodopseudomonas* with natural organic matter decomposition, although colored microbial mats have been found to occur in swine wastewater ditches where PNSB dominate (Okubo et al., 2006) and in red water with purple anoxygenic phototrophic bacterial blooms (Belila, Gtari, Ghrabi, & Hassen, 2009).

Before the experiment reported in this paper, we attempted to obtain organic and inorganic nutrients from fish feed decomposition to study *Microcystis* blooms. In 2014, fish feed and tap water were placed in tanks without aeration. After slow degradation during the first few weeks, the fish feed remained relatively stable, as the dissolved oxygen in the water was consumed, and the feed remained unchanged in an almost anaerobic environment. To promote the decomposition of fish feed, we used aerated tanks starting from January 2015. During warm periods in 2015, we observed some interesting phenomena in the tanks. Some dark red material attached to the inner walls of the tanks, and the water in the tanks also gradually became dark red. Analysis of the dark red material and the dark red water revealed that the color was due to the dominance of *Rhodopseudomonas*. On the basis of these observations, we carried out further experiments to test and verify the ability of *Rhodopseudomonas* to be cultured in the presence of fish feed after decomposition produces sufficient nutrients.

Therefore, the aim of the current study was to determine (a) whether the dark red microbial growth in the presence of decomposing fish feed was a random occurrence; (b) whether the culture method could be replicated; and (c) if the phenomenon was a regular occurrence, then what microbial changes occur during the cultivation process, and what factors stimulate these changes.

We reasoned that if the results of this study indicated that both attached and suspended dark red microbial growth could be achieved by using compound fish feed decomposition in transparent containers, then this research might provide a technical reference for the use of organic waste such as livestock manure, concentrated cyanobacterial blooms, and food waste. These waste products could be converted to high densities of *Rhodopseudomonas*, and the bacteria could then be used in aquaculture water quality control, animal feed additives, or hydrogen production.

## 2 MATERIALS AND METHODS

### 2.1 Experimental setup and management

From 5 January 2015 to 31 May 2016, extruded freshwater compound fish feed was used as the base material for *Rhodopseuodomonas* culture in a 220 m^2^ greenhouse. The greenhouse was located in Yangpu District, Shanghai, China (N 31°16′52.75″, E 121°29′46.88′′). The air temperature in the greenhouse reached up to 45°C with some windows open during the hot summer and decreased to as low as 5°C with all windows closed during the cold winter. The frame of the greenhouse decreased the light intensity by approximately 50%.

Eight transparent containers (TK1–TK8) were filled with tap water and fish feed for the culture process; the details are provided in Table 1. The ratio of fish feed to tap water was 14.3–50.8 g/L. During the incubation, no other substances or strains were added. The compound fish feed was composed of high‐quality fish meal, soybean meal, animal protein, yeast powder, flour, calcium dihydrogen phosphate, trace elements, vitamins, and amino acids; the content of crude protein, calcium dihydrogen phosphate, and trace elements was 35.0%, 1.0%, and 0.3%, respectively.

**Table 1:** The culture conditions in containers TK1–TK8

Container	Container characteristics	Start date (mm/dd/yyyy)	Amount of compound fish feed added (kg)	Total volume of tap water and fish feed (L)	Ratio of fish feed to tap water (g/L)	Aeration management	Earliest time required for almost full inner wall attachment of dark red material (d)
TK1	Transparent borosilicate glass tank (60 cm × 35 cm × 40 cm)	12/01/2015	1.0	70	14.3	Continuous	45–60
TK2	Transparent borosilicate glass tank (60 cm × 35 cm × 40 cm)	12/01/2015	1.0	70	14.3	Continuous	70–80
TK3	Colorless transparent plexiglas cylinder (40 cm diameter)	01/05/2015	5.0	130	38.5	The initial 6 months (approximately)	120–140
TK4	Colorless transparent plexiglas cylinder (40 cm diameter)	09/08/2015	6.6	130	50.8	Continuous	80–90
TK5	Colorless transparent plexiglas cylinder (40 cm diameter)	09/08/2015	3.3	130	25.4	Continuous	90–100
TK6	Colorless, transparent borosilicate glass conical flask (5 L)	2/23/2016	0.15	6.5	23.1	The initial 3 months	110–120
TK7	Colorless, transparent borosilicate glass conical flask (5 L)	07/11/2016	0.15	5.5	27.3	The initial 2 months	95–110
TK8	Transparent borosilicate glass tank (60 cm × 35 cm × 40 cm)	07/15/2015	3.0	70	42.9	Continuous	95–110

The process in two of the eight tanks (TK1 and TK2, 60 cm × 35 cm × 40 cm) is described below. On 1 December 2015 (winter in Shanghai, China), 1.0 kg of compound fish feed was added to TK1 and TK2. Tap water was then added to the tanks, to a total volume of 70 L.

Tanks TK1 and TK2 were heated to 33–35°C with a 300 W heating rod from the start of the experimental period until day 64 (3 February 2016). During the culture process, the heating rod became damaged by the growth of fouling organisms. After the old heating rod stopped functioning properly, it was replaced with a new heating rod. From day 64 onward, heating was stopped, and the culturing process continued at ambient temperature.

Tanks TK1 and TK2 were continuously aerated with an air stone, which was set at the bottom of each tank. The aeration strength was adjusted to avoid any foam overflow in the tank. Aeration provides air, particularly oxygen, which is required for fish feed decomposition. During the culturing process, tap water was replenished approximately every 3 days to restore the total volume in the tank to 70 L.

The culturing process in TK3–TK8 was very similar to that for TK1 and TK2, and was performed in the same greenhouse under the same ambient temperature. Further details are presented in Table 1.

### 2.2 Water quality sampling and analysis

During the culturing process, water samples were collected for determination of total nitrogen (TN), total phosphorus (TP), and total dissolved carbon (TOC) from TK1 and TK2 between 09:00 and 10:00 approximately every 15‒20 days. Measurements of TN and TP were performed according to the methods of Gross and Boyd (Gross & Boyd, 1998), and TOC content was determined with a TOC analyzer (MultiN/C UV, Analytik Jena Inc.). The pH values, dissolved oxygen (DO), and salinity were measured in situ in the middle of the tanks with a handheld multiparameter meter (Professional Plus, YSI).

### 2.3 Microbial community analysis

#### 2.3.1 Microbial sample collection

During the experiment, similar phenomena occurred in tanks TK1 and TK2. However, the water color in TK1 changed faster than that in TK2; therefore, microbial samples were collected in tank TK1. Suspended bacterial samples were collected in TK1 on days 10, 34, 70, and 142, and the attached bacteria were sampled on days 55 and 70 when the inner wall was dark red or red. The water color in TK1 was dark brown with a reddish tinge on day 70 and became dark brown on day 142. A 0.22‐μm filter (Millipore) was used to collect the bacterial cells.

#### 2.3.2 DNA extraction and PCR amplification

After the bacterial cells were collected with 0.22‐μm filters, DNA was extracted separately with an E.Z.N.A.^®^ soil DNA Kit (Omega Bio‐tek) according to the manufacturer's protocols. The final DNA concentration and purity were determined with a NanoDrop 2000 UV‐Vis spectrophotometer (Thermo Scientific), and DNA quality was verified with 1% agarose gel electrophoresis.

The diversity of bacterial plankton was determined by analysis of the V4–V5 hypervariable regions of the 16S rRNA gene, which was amplified with the primer set 515F (50‐GTGCCAGCMGCCGCGGTAA‐30) and 907R (50‐CCGTCAATTCMTTTRAGTTT‐30) with a thermocycler PCR system (GeneAmp 9,700, ABI). The PCRs were conducted with the following program: 3 min of denaturation at 95°C, 27 cycles of 30 s at 95°C, 30 s annealing at 55°C, 45 s elongation at 72°C, and a final extension at 72°C for 10 min.

PCR was performed in triplicate with a 20 μl mixture containing 4 μl of 5 × FastPfu Buffer, 2 μl of 2.5 mM dNTPs, 0.8 μl of each primer (5 μM), 0.4 μl of FastPfu Polymerase, and 10 ng of template DNA. The PCR products were extracted from a 2% agarose gel and further purified with an AxyPrep DNA Gel Extraction Kit (Axygen Biosciences) and quantified with a QuantiFluor™‐ST fluorometer (Promega) according to the manufacturer's protocol.

#### 2.3.3 Illumina MiSeq sequencing

Purified amplicons were pooled in equimolar amounts and subjected to paired‐end sequenced (2 × 300) on an Illumina MiSeq platform (Illumina) according to the standard protocols by Majorbio Bio‐Pharm Technology Co. Ltd.

#### 2.3.4 Processing of sequencing data

Raw fastq files were demultiplexed, quality‐filtered with trimmomatic, and merged with FLASH with the following criteria: (a) The reads were truncated at any site receiving an average quality score <20 over a 50 bp sliding window. (b) Primers were exactly matched, allowing 2 nucleotide mismatches, and reads containing ambiguous bases were removed. (c) Sequences whose overlap was longer than 10 bp were merged according to their overlap sequence.

Operational taxonomic units (OTUs) were clustered with a 97% similarity cutoff in UPARSE (version 7.1 http://drive5.com/uparse/), and chimeric sequences were identified and removed with UCHIME (Edgar, 2013). The taxonomy of each 16S rRNA gene sequence was analyzed with the RDP Classifier algorithm (http://rdp.cme.msu.edu/) (Wang, Garrity, Tiedje, & Cole, 2007) against the Silva (SSU128) 16S rRNA database, with a confidence threshold of 70%.

According to cluster information, alpha diversity parameters referring to species richness (Chao), community diversity (Shannon index), and sequencing depth (Good's coverage) of the bacterial community were analyzed from 16S rRNA gene amplicon sequence data with MOTHUR software (http://www.mothur.org).

### 2.4 Microbial biomass analysis

Microbial biomass analysis was conducted on the microbial samples collected in tank TK1, including the suspended samples from day 70 and attached samples from days 55 and 70. The microbial biomass was expressed as wet weight and dry‐cell weight. Wet weight was changed to dry‐cell weight by drying in an oven at 100°C for 12 hr (Kim & Lee, 2000).

For suspended cell biomass analysis, the conversion to wet weight biomass was based on the assumption that 1 mm^3^ of volume was equivalent to 1 mg of wet weight biomass. Bacterial volumes were calculated from cell density and cell size measurements. At least 50 bacterial units were measured to obtain the mean cell size. The number of dark red bacteria was calculated in 0.1 ml counting chambers with two replicate aliquots, which were observed at ×1,000 magnification by light microscopy with oil immersion objective and analyzed through phytoplankton biomass counting methods (Wang, Qin, Gao, & Paerl, 2010).

In attached cell biomass analysis, a specific area of the attached bacterial layer was scraped down to obtain the wet weight. For this process, a thick area with a smooth surface was selected. Care was taken to ensure that as few crystallized salt grains as possible were mixed during this operation.

### 2.5 Inorganic salt crystal collection and analysis

During the culture, some clear and colorless crystals appeared both on the inner walls of the culture tanks and the heating rods. The inorganic salt that had crystallized on the heating rod in TK1 was collected on 27 January 2016 to perform elemental analysis through an Axios X‐ray fluorescence spectrometer (PANalytical B.V.). The thickness of the attached bacteria was measured with Digimatic calipers (Mitutoyo) after some water was discharged from the tanks.

## 3 RESULTS

### 3.1 Observations during the culture process

The water color changed as the fish feed decomposed. The variations in water color in each experimental tank were similar. As the fish feed decayed, the water gradually became pale yellow and odorous; then, the water gradually became pale gray before becoming brown or black brown. Subsequently, the water became reddish brown or even dark red, and the inner walls of the containers became dark red before the water inside the tank became dark red. After becoming dark red, the water gradually became dark brown and then light brown.

**Figure 1:**
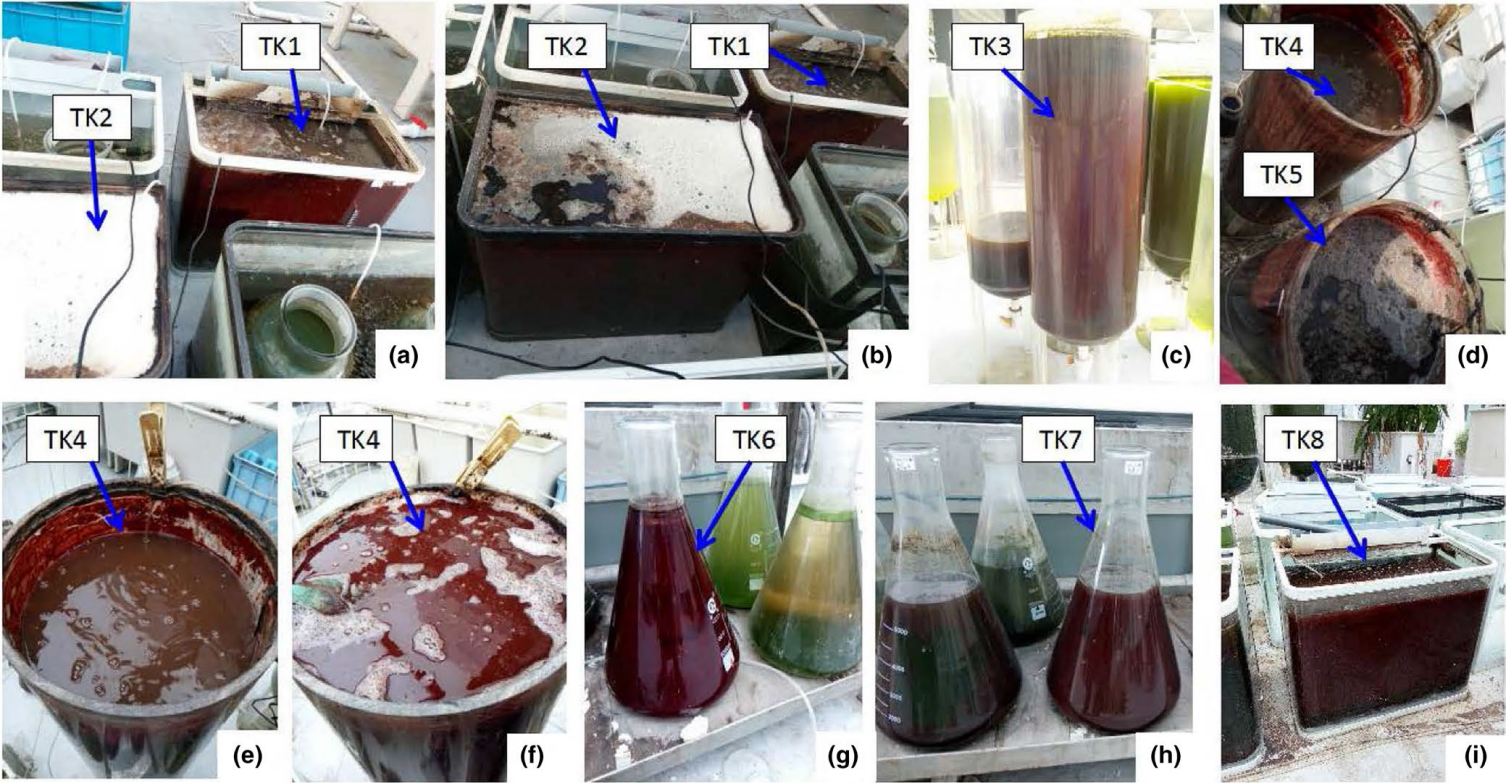
Photographs of tanks TK1–TK8 with dark red bacteria attached on their inner walls during the culture. a: tanks TK1 and TK2 on day 55; b: tanks TK1 and TK2 on day 60; c: tank TK3 on day 191; d: TK4 and TK5 on day 80; e: TK4 with part of the water removed on day 96; f: tank TK4 on day 127; g: tank TK6 on day 139; h: tank TK7 on day 115; i: tank TK8 on day 118

Similar results were achieved in all containers, with dark red material becoming attached to the inner walls and the water then turning dark red. When the inner walls were dark red, the thickest attached layer of the inner walls was approximately 0.5‒1.0 mm in containers TK1–TK8. Figure 1 shows photographs of containers TK1–TK8 with the attached dark red bacteria on their inner walls.

### 3.2 Microorganisms

A total of 262,302  high‐quality sequences were obtained by analyzing the six microbial samples from container TK1, which belonged to 609 OTUs (UPARSE at 97% cutoff), 476 species, 346 genera, 166 families, 90 orders, 46 classes, and 23 phyla.

The alpha diversity index and the library coverage of the six samples are shown in Table 2.

**Table 2:** The alpha diversity index and library coverage of the six microbial samples from container TK1

Suspended samples	Attached samples	OTUs	Chao	Shannon	Coverage
Day 10		209	290.05	3.74	0.994
Day 34		91	140.40	1.24	0.996
Day 70		212	269.12	2.84	0.996
Day 142		310	352.77	3.59	0.998
	Day 55	171	218.05	0.68	0.999
	Day 70	138	205.56	1.01	0.998

**Figure 2:**
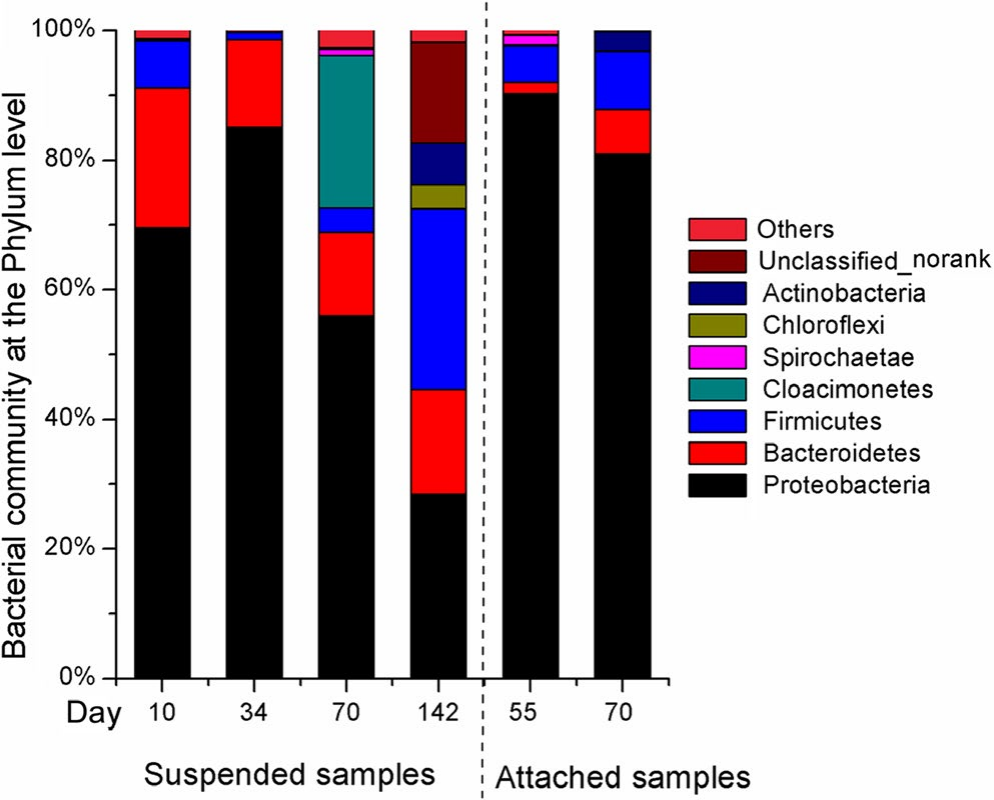
The relative abundance (%) of the bacterial community at the phylum level from the six microbial samples from container TK1

Figure 2 shows the relative abundance of bacterial communities in the six samples at the phylum level. Among all detected taxa, Proteobacteria accounted for 70.1% of all OTUs and were the most dominant. Firmicutes and Bacteroidetes were less dominant.

**Figure 3:**
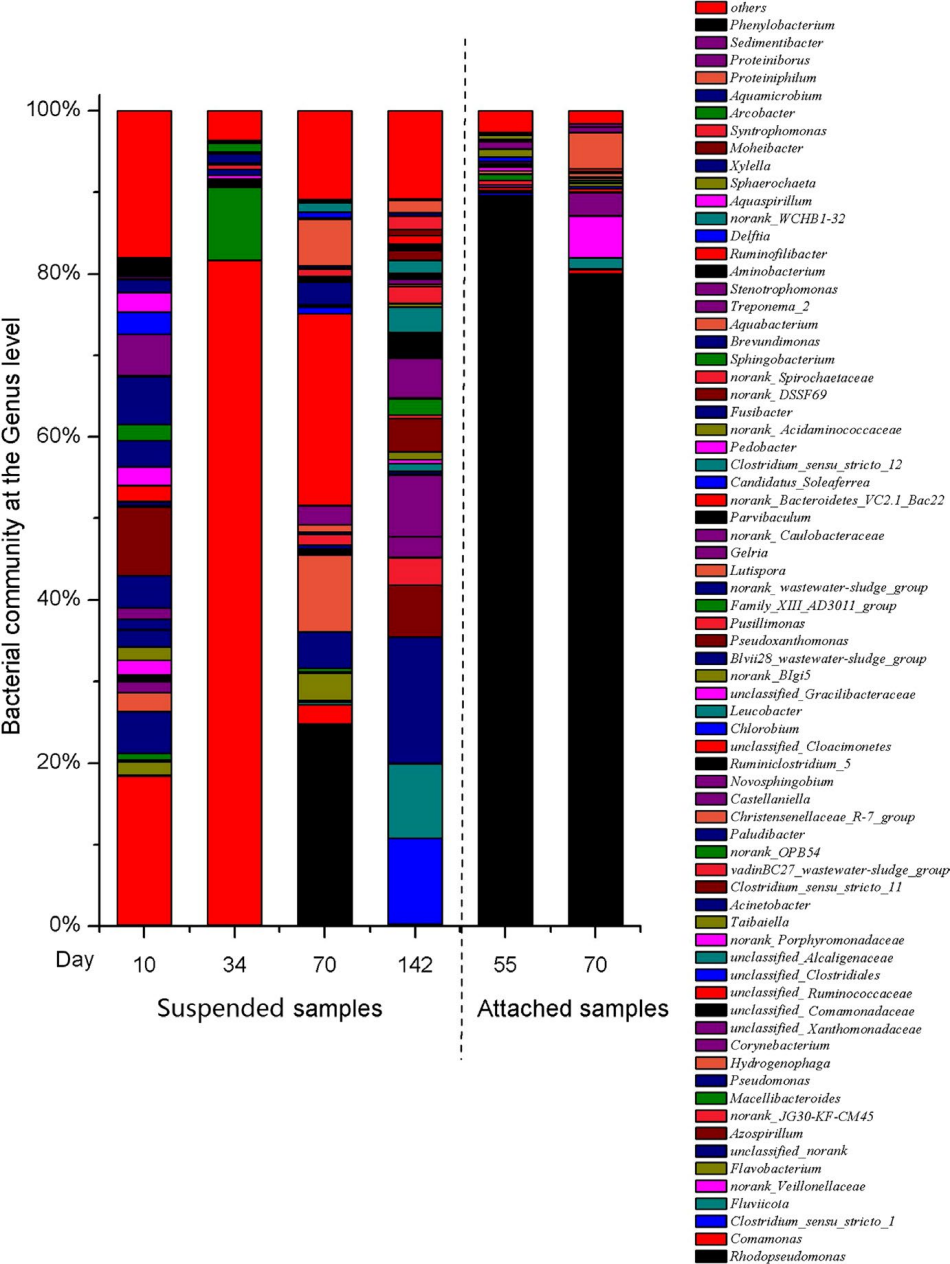
The relative abundance (%) of the bacterial community at the genus level from the six microbial samples from container TK1

Figure 3 shows the bacterial community at the genus level in the six samples. Changes were observed in the dominant genus during the culturing process. *Comamonas* was dominant on day 10 and accounted for 18.3% of the OTUs. The following genera were also identified: *Pseudoxanthomonas*, *Pseudomonas*, *Fusibacter*, *Delftia,* and *Phenylobacterium*, accounting for 8.4%, 5.2%, 3.3%, 2.7%, and 2.3% of the OTUs, respectively. *Comamonas* accounted for 81.4% of the OTUs on day 34 and mainly comprised *C. denitrificans*; the next most dominant genera were *Macellibacteroides* and *Arcobacter*, accounting for 9.0% and 1.1% of the OTUs, respectively.

*Rhodopseudomonas* dominance in the suspended samples was reached on day 70, and this is genus accounted for 24.8% of the OTUs. The following genera were unclassified: Cloacimonetes, *Hydrogenophaga*, *Aquabacterium*, *Pseudomonas,* and *Flavobacterium*, which accounted for 23.5%, 9.4%, 5.7%, 4.4%, and 3.4% of the OTUs, respectively.

The most dominant genus in attached samples on days 55 and 70 was *Rhodopseudomonas*, which accounted for 89.6% and 80.0% of the OTUs, respectively. Then on day 142, *Clostridium *sensu* stricto* 1, *Fluviicola*, unclassified genus, unclassified Xanthomonadaceae, and *Castellaniella* became dominant, accounting for 10.5%, 9.1%, 15.5%, 7.5%, and 4.8% of the OTUs, respectively.

*Rhodopseudomonas faecalis* was the main *Rhodopseudomonas* species in both the suspended and attached samples.

The dark red bacteria were dominated by rod‐shaped cells. The dark red cells were 0.5–0.8 μm in width and 1.0–2.5 μm in length. Microbial biomass analysis indicated that the cell density of the suspended dark red bacteria on day 70 was 1.983 ×1.0^12^ , with a dry‐cell weight of 0.056 g/L, and the dry‐cell weight of the thickest layer on days 55 and 70 was 6.86 and 7.50 mg/cm^2^, respectively (Table 3).

**Table 3:** Density, weight, and thickness of the dark red bacterial biomass of suspended samples on day 70, and attached samples on days 55 and 70 of the experiment

Suspended samples	Attached samples	Cell density (cells/L)	Wet weight (g/L or mg/cm^2^)	Dry‐cell weight (g/L or mg/cm^2^)	Attached layer thickness (mm)
Day 70		1.983 × 1.0^12^	0.701	0.056	–
	Day 55	–	58.0	6.86	0.48
	Day 70	–	67.2	7.50	0.56

Notes

The weight unit “g/L” is for suspended bacteria, and “mg/cm^2^” is for attached bacteria.

### 3.3 Water quality in TK1 and TK2

**Figure 4:**
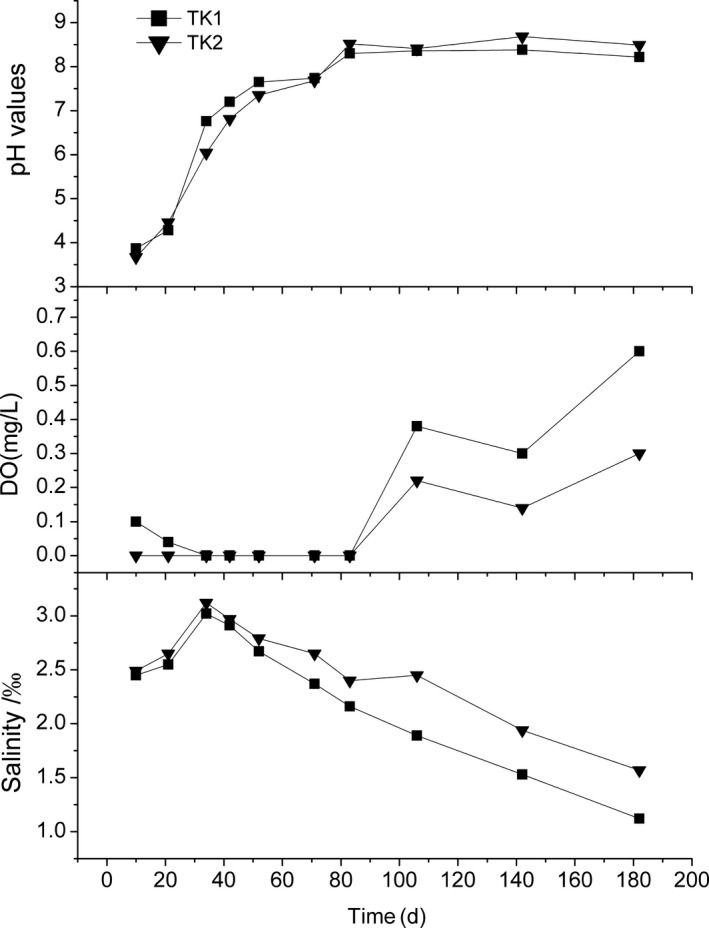
Changes in pH, DO, and salinity levels in TK1 and TK2

As seen in Figure 4, the pH value on day 10 was approximately 3.7 and increased to approximately 7 on day 40, 8 on day 80, then subsequently increased to approximately 8.5. The DO was nearly 0 mg/L from the start of the culture period until day 83, after which it increased to 0.14–0.6 mg/L. The salinity was approximately 2.5 % on day 10, increased to 3.0‰ on day 42, and subsequently decreased.

The TN, TP, and TOC levels in TK1 and TK2 (Table 4) were highest on day 57, and those in TK1 were lower than those in TK2. The highest TN, TP, and TOC values in TK2 (on day 57) were approximately 730 mg/L, 356 mg/L, and 1,620 mg/L, respectively.

**Table 4:** The TN, TP, and TOC content in the water from TK1 and TK2 during the culture period (mg/L)

Day	Date (mm/dd/yyyy)	TK1			TK2		
		TN	TP	TOC	TN	TP	TOC
10	12/11/2015	542.42	316.45	901.8	613.54	278.73	1,276.6
34	01/04/2016	560.38	334.65	1,101.2	642.51	298.86	1,418.6
57	01/27/2016	632.57	356.42	1,246.4	730.42	327.54	1,620.6
87	02/26/2016	425.42	217.45	776.8	546.55	236.76	1,015.3
106	03/16/2016	336.12	137.33	436.2	408.12	156.53	625.7
142	04/21/2016	257.5	123.87	283.4	296.33	110.65	334.6
182	05/31/2016	100.33	43.87	150.1	144.23	53.56	172.0

**Figure 5:**
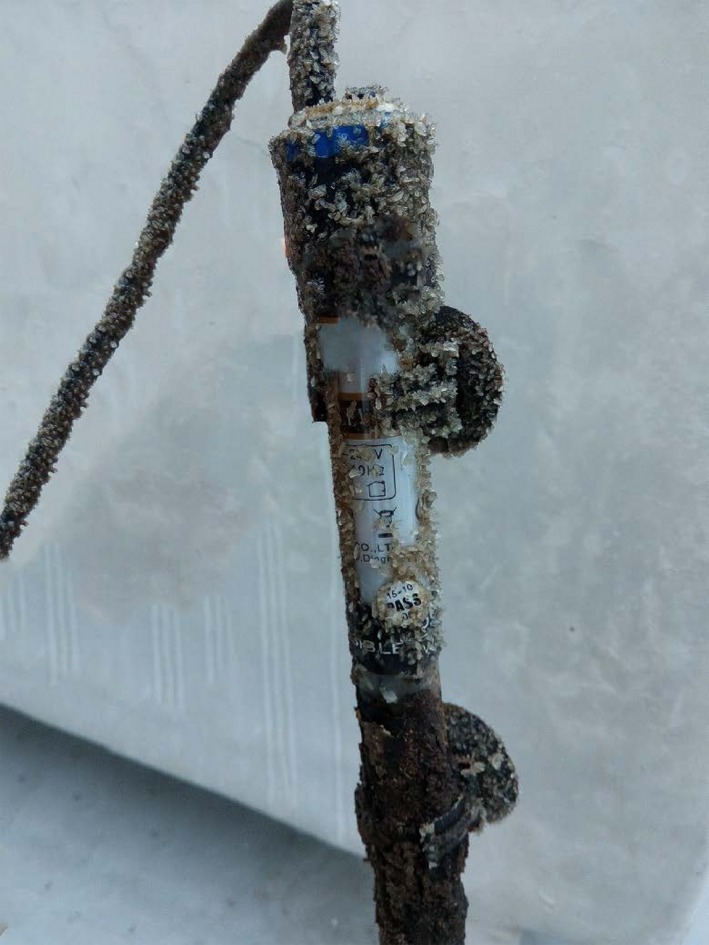
The inorganic salt crystallized on the heating rod in TK1 on day 57 (27 January 2016)

Some inorganic salt was crystallized on the inner walls and the heating rod of TK1 (Figure 5). X‐ray fluorescence spectrometry analysis indicated that the main elements present in the salt were O, P, Mg, C, and N (Table 5). However, the light element hydrogen cannot be measured by this method, and substantial amounts of compounded hydrogen were likely to be present in the salt crystals.

**Table 5:** The elemental composition of the crystallized inorganic salt from TK1 on 27 January 2016

Element	Mass percentage (%)	Molar ratio from mass percentage divided by relative atomic mass
O	49.8396	3.1151
P	21.5298	0.6951
Mg	14.6776	0.6039
C	7.5935	0.6322
N	4.6459	0.3317
Si	0.5655	0.0201
Ca	0.5512	0.0138
K	0.3124	0.0080
Al	0.1228	0.0046
S	0.0962	0.0030
Fe	0.0351	0.0006
Mn	0.0304	0.0006

The crystallized inorganic salt was able to partially dissolve in water, and some ammonium and phosphate radicals were measured in the dissolved solution, thus indicating that the salt may have contained more than one compound. On the basis of some calculations using the elemental composition of the crystallized inorganic salt (Table 5), we speculated that the salt might be composed of magnesium carbonate (MgCO_3_) and di‐ammonium hydrogen phosphate ([NH_4_]_2_HPO_4_), as well as struvite (MgNH_4_PO_4_.6H_2_O).

## 4 DISCUSSION

Figure 1 shows that containers TK1–TK8 with different fish feed loading (Table 1) showed dark red bacteria attached to their inner walls. The dominance of *R. faecalis* in both the suspended samples on day 70 and attached samples on days 55 and 70 (Figure 3) indicated that *Rhodopseudomonas* was dominant both in the water and on the container walls. The dark red color in the containers was caused by the presence of the photosynthetic bacterium *Rhodopseudomonas*.

The results of the culture process in TK1‐TK8 were similar. Observations during the culture period revealed that dark red bacteria gradually attached to the inner walls of the containers and the water turned dark red, despite the fact that the culture conditions in the eight containers differed in terms of fish feed loading, water temperature ranges, and light intensity. The results showed that *Rhodopseudomonas* was inclined to dominate in both the attached and suspended forms during the culture process with the ratio of fish feed to tap water ranging from 14.3 to 50.8 g/L in transparent containers.

Before the dominance of *Rhodopseudomonas* during the culturing period, *Comamonas denitrificans* was dominant on both days 10 and 34 (Figure 3). *Pseudoxanthomonas*, *Pseudomonas,* and *Fusibacter* were also dominant on day 10 (Figure 3). These dominant genera are very common in the decomposition of organic materials (Andersson, Rajarao, Land, & Dalhammar, 2008; Ben et al., 2012; Gumaelius, Magnusson, Pettersson, & Dalhammar, 2001; Kumar, Revathi, & Khanna, 2015). *C. denitrificans* is a Gram‐negative, oxidase‐, and catalase‐positive bacterium with efficient denitrifying ability, which can reduce nitrate to nitrogen gas (Gumaelius et al., 2001). *C. denitrificans* 123 is found in wastewater treatment systems, and it has been reported to form pure culture biofilms in four different culture media (Andersson et al., 2008). *Pseudoxanthomonas* is a Gram‐negative, aerobic, heterotrophic bacterium, which can degrade cellulose and lignocellulose (Kumar et al., 2015). *Pseudomonas* is ubiquitous in soil and water, and *Fusibacter* is a minor genus within the Firmicutes phylum, which mainly comprises four Gram‐positive species with valid published names (Ben et al., 2012). *Fusibacter* was first recorded with the discovery of the thiosulfate‐reducing bacterium *Fusibacter paucivorans* (Ravot et al., 1999). One of the species, *Fusibacter tunisiensis*, has been used to treat olive‐mill wastewater (Ben et al., 2012).

*Rhodopseudomonas faecalis* dominance in both the suspended and attached samples was achieved during the culturing period (Figure 3). The name *R. faecalis* was proposed for a new species of the genus *Rhodopseudomonas*, which was isolated from an anaerobic reactor digesting chicken feces (Zhang et al., 2002) and showed a strong tendency for photoorganotrophy with simple organic compounds as electron donors and carbon sources. Thus, during the decomposition of compound fish feed in transparent containers, *R. faecalis* was dominant in both the suspended and attached forms with high biomass (Table 3), thus indicating that this method can be used for *R. faecalis* culture.

Many factors, such as oxygen, water temperature, light, substrate composition, and substrate concentration, affect PPB community composition, particularly in the case of *Rhodopseudomonas* culture.

Oxygen is an important factor affecting the occurrence of PPB. *Rhodopseudomonas palustris* has been reported to be much more tolerant of oxygen than previously believed (Chandaravithoon et al., 2018). Some PNSB species can oxidize under light, microaerobic, or aerobic conditions (Garrity, Boone, & Castenholz, 2001). In the current experiment, the culture tanks were continuously aerated with air stones to improve the feed degradation, although the measured DO was nearly 0 mg/L during the initial 90 days (Figure 4). The aeration process can cause part of the feed material to become suspended in the water column, thus enabling the feed material to come into contact with the aerated oxygen. The dominance of denitrifying *C. denitrificans* on both days 10 and 34 (Figure 3) showed that some oxygen was already present in the system. With the degradation of the organic matter from the fish feed, the process of organic decay consumed less oxygen, resulting in a gradual increase in DO levels in the water (Figure 4).

Water temperature can affect the growth of *Rhodopseudomonas*. In this experiment, the time for attached *Rhodopseudomonas* to become dominant changed from approximately 45 to 140 days (Table 1), across two or more different seasons with different water temperature ranges. Similarly, Zhang et al. (2002) have reported that *R. faecalis* can grow at temperatures ranging from 28 to 45°C. Water temperature, in addition to the concentrations of some nutrients, is the main parameter controlling *Rhodobacter* sp. strain PS9 blooms (Do et al.., 2003). As TK1 and TK2 were heated to 33‒35°C from the start of the experiment until day 64, feed decomposition occurred rapidly, and the dominance of *Rhodopseudomonas* occurred more quickly than that in other tanks (Table 1). This result indicates that suitable high temperatures are conducive to the rapid growth of the bacteria.

Light is also an important factor affecting the PPB community. PNSB are photoheterotrophs capable of photoautotrophic nutrition and can be grown effectively in the dark (Madigan & Jung, 2009). The photoheterotrophic growth mode is normal for PNSB (Venkidusamy & Megharaj, 2016). Aerobic growth of *R. palustris* strain RP2 is possible under both illuminated and nonilluminated conditions (Venkidusamy & Megharaj, 2016). However, some researchers have found that some strains of *Rhodopsedumonas* such as *R. faecalis* (Zhang et al., 2002) and *R. palustris*‐17 (Dönmez, Öztürk, & Çakmakçi, 1999) are unable to grow in oxygen‐rich, illuminated environments. In the current experiment, the attached *Rhodopseudomonas* grew on the inner wall of the container, where the frame of the greenhouse decreased the light intensity by approximately 50%, demonstrating that the attached bacteria were photoheterotrophic. The containers in this experiment were transparent, and the provision of light is an important factor in achieving growth of attached *Rhodopseudomonas*.

Previous investigations have shown that substrate loading is a key parameter in determining biofilm structure and function (Wijeyekoon, Mino, Satoh, & Matsuo, 2004). Peyton (1996) has found that *Pseudomonas aeruginosa* biofilm thickness increases with the substrate loading rate. Substrate and hydrodynamic conditions significantly influence the structure, density, thickness, and substrate conversion rate of the resulting biofilm (Wäsche, Horn, & Hempel, 2002). *R. faecalis* strain A isolated from swine sewage wastewater has been found to grow in twice diluted pig farm effluent of 4,575 mg/L TOC and 693 mg/L ammonia nitrogen, in which it enters the logarithmic growth phase after 48 hr of incubation (Wei et al., 2016). *Rhodopseudomonas faecalis* PA2 has also been cultivated in sterilized domestic wastewater of approximately 176 mg/L total Kjeldahl nitrogen and 121 mg/L total phosphate, with inoculation of 10% in different light intensities and agitation speeds (Saejung & Ampornpat, 2017). The bacteria grow rapidly within 10 days (Saejung & Ampornpat, 2017) but do not form a biofilm on the inner walls of the containers during that time.

Research (Hülsen et al., 2016) similar to the current study has been carried out, in which domestic wastewater was collected and used as reactor feed, and a minimum of 200 mg/L of ethanol was supplied, with no inoculum addition. In that experiment (Hülsen et al., 2016), the reactor biomass color became purple within 22 days, and *Rhodocyclus* was replaced with *Rhodopseuodomonas* and *Rhodobacter* from day 115. The *Rhodopseuodomonas* abundance was 60% at day 193 (Hülsen et al., 2016), and during the culture process, the concentration of total Kjeldahl nitrogen and total phosphorus in the wastewater was 60 ± 11–63 ± 8 mg/L and 8.6 ± 1.8–8.7 ± 1.6 mg/L, respectively (Hülsen et al., 2016). However, there was no clear biofilm formation on the reactor walls. In the current study, the highest levels of TN, TP, and TOC in TK1 and TK2 were approximately 730 mg/L, 356 mg/L, and 1,620 mg/L, respectively (Table 4), values much higher than those reported by (Hülsen et al., 2016). Wijeyekoon et al. (2004) have found that biofilm growth rate is positively influenced by substrate loading. Given the results of the current study, we suggest that the nitrogen and phosphorus substrate concentrations of the reactor in Hülsen et al. (2016) may have been too low to produce attached *Rhodopseuodomonas*.

Numerous reports have described the culture of attached bacteria in biofilms, because attached growth can be highly productive, and attached growth, in comparison with suspended growth, makes bacterial harvesting much easier. Biofilms with diverse aerobic and anaerobic microbial communities play important roles in wastewater treatment (Fernández, Díaz, Amils, & Sanz, 2008). Biofilms with high cell densities can be formed in batch biogas reactors, both at high and low organic loading rates, with anaerobic digestion to digest organic waste (Langer, Schropp, Bengelsdorf, Othman, & Kazda, 2014). However, the dominant bacterial species were not reported in Langer et al. (2014). The natural attachment methods occurring during the formation of biofilm promote maximum cell viability and biochemical activity after attachment (Robledo‐Ortíz et al., 2010). Improving surface attachment for bacteria is considered to be a reasonable approach for photofermentation H_2_ production with *R. faecalis* RLD‐53 (Xie et al., 2012).

Increasing the biomass of *Rhodopseudomonas* has been the goal of many studies, because biomass is very important in the application of *Rhodopseudomonas*, including the production of carotenoids, animal feed additives, or hydrogen. In this experiment, the cell density and biomass of suspended *Rhodopseudomonas* on day 70 (Table 3) were lower than that reported in several previous studies (Kim & Lee, 2000; Saejung & Ampornpat, 2017). Kim and Lee (2000) have reported that the maximum number of viable cells is 2.65 g/L of dry‐cell weight in batch fermentation with a modified MYC medium. Maximum biomass production can be as high as 33.9 g/L in domestic wastewater (Saejung & Ampornpat, 2017). However, the thickness of the attached bacterial layer in the current study was greater than those studies (Liao et al., 2013; Liao, Zhong, Zhu, Huang, & Chen, 2015; Wang, Liao, Wang, Zhu, & Li, 2011), although the culture process was longer in the current study. Wang et al. (2011) have reported that the dry weight and thickness of *Rhodopseudomonas* biofilms are approximately 1.0 mg/cm^2^ and 0.02 mm, respectively. The highest value for the dry weight of the biofilms has been reported to be approximately 0.8 mg/cm^2^ (Liao et al., 2013, 2015), and the thickness of the biofilms has been reported to be as high as 0.18 mm in a synthetic medium (Liao et al., 2015). Therefore, the methods used in the current study produced an increased biomass of attached *Rhodopseudomonas*.

Photosynthetic bacteria are highly metabolically diverse. *Rhodopseudomonas* can use a wide range of compounds such as sugar, low fatty acids, organic acids, and mixed alcohols as carbon sources (Basak, Jana, & Das, 2016), and some *Rhodopseudomonas* spp. are able to grow anaerobically in the light or aerobically in the dark with many carbon sources and electron donors (Zhang et al., 2002). *Rhodopseudomonas palustris* strain RP2 is capable of mineralizing diesel range hydrocarbons in anaerobic environments (Hülsen et al., 2016). Although the composition of the materials from the degradation of compound fish feed was not analyzed in this study, the pH values at the early stage of degradation may be as low as 3.7, and those during the initial 40 days were always less than 7.0 (Figure 4), thus indicating that some acids were produced during the degradation of fish feed. Similarly, the presence of oxygen allows for the proliferation of acetic acid bacteria to produce high concentrations of acetic acid during water kefir fermentation (Hülsen et al., 2016), whereas high concentrations of short‐chain fatty acids promote the dominance of PNSB (Hiraishi, 1991; Okubo et al., 2005). The bioavailability of lower fatty acid concentrations in wastewater is a key factor allowing the formation of visible microbial mats with dominance of *Rhodobacter* and *Rhodopseudomonas*: Members of the genus *Rhodopseudomonas* have a wider spectrum of lower fatty acids utilization than those of the genus *Rhodobacter* (Okubo et al., 2006).

The composition of the synthetic culture medium for *Rhodopseuodomonas* (Weaver et al., 1975) showed that metal cations and trace elements, as well as nitrogen, phosphorus, and carbon sources, are necessary factors for *Rhodopseuodomonas* growth. In this experiment, some crystallized salt appeared, and ammonium and phosphate radicals were measured in the dissolved solution of the crystallized salt, thus indicating that the water from the decomposition of fish feed was high in dissolved nitrogen and phosphorus nutrients. In addition, the TN content, TP content, and TOC content in the water during the culture period were high (Table 4). Moreover, the elemental composition of the crystallized salt (Table 5) indicated the presence of many metal cations in the water, supplying metal cations and some trace elements. The compound fish feed also contained trace elements. Thus, the metal cations and trace elements, in addition to the high content of nitrogen, phosphorus, and carbon, in the eutrophic water (Table 4) facilitated the fast growth of attached and suspended *Rhodopseuodomonas*. In particular, the attachment of salt to the inner tank wall is an important factor promoting the formation of a thick bacterial layer, by providing a persistent and stable substrate for mass growth.

There was an increase in pH and DO levels in TK1 and TK2, with a decrease in salinity levels (Figure 4). Some crystallized inorganic salt formed during the culture process (Figure 5), which was likely composed of magnesium carbonate (MgCO_3_) and di‐ammonium hydrogen phosphate ([NH_4_]_2_HPO_4_), as well as struvite (MgNH_4_PO_4_.6H_2_O). The ions in the water were likely NH_4_^+^, Mg^2+^, PO_4_^3−^, and CO_3_^2−^. With the decay of the fish feed and the growth of bacteria, DO and pH values increased in particular after 90 days, while the concentration of NH_4_^+^ decreased due to aerobic bacteria activity. At the same time, the increased pH decreased the solubility of struvite, which was influenced by many parameters including pH value (Le Corre, Valsami‐Jones, Hobbs, & Parsons, 2009). Borgerding (1972) reported that struvite solubility could decrease from around 3,000 mg/L to less than 100 mg/L with an increase in pH from 5 to 7.5, while Buchanan, Mote, and Robinson (1994) identified pH 9 as the minimum pH required for struvite solubility. As the pH value increased to about 8.5 in this experiment (Figure 4), the struvite solubility likely decreased. Thus, the salinity decreased gradually after 40 days.

## 5 CONCLUSION

In conclusion, this study provides details of a method for the mass culture of *Rhodopseudomonas* in the presence of compound fish feed. This method involves placing compound fish feed and tap water into aerated transparent containers. The ratio of fish feed to tap water was 14.3‒50.8 g/L. No other inoculum enrichment or substances were needed during the culture process. As the fish feed decomposed, high nutrient concentrations, including high levels of nitrogen, phosphorus, carbon, and metal cations, were achieved, thus allowing both attached and suspended *Rhodopseudomonas* to become dominant from days 45 to 120 of the experiment. The biomass of attached *Rhodopseudomonas* attained in the current study is higher than that reported in many other studies. This method can facilitate large‐scale cultivation of *Rhodopseudomonas* spp. by using aquatic fish feed fermentation.

## CONFLICT OF INTERESTS

None declared.

## AUTHOR CONTRIBUTIONS

XW performed the experiments, analyzed data, and contributed to drafting the manuscript. XL analyzed data, participated in the preparation of the manuscript, and analyzed the results. SL performed some experiments, analyzed data, and contributed to drafting the manuscript. CL, ZG, and XZ participated in the experiments and analyzed the results. QN analyzed the results. All authors have approved the final version of the manuscript.

## ETHICS STATEMENT 

None required.

## Data Availability

The sequence data have been deposited in the Sequence Read Archive (SRA) under accession number SRP190363.
